# Supplementary material for: Bacterial Symbionts in Ceratitis capitata
Source: Insects. 2022 May 19;13(5):474. doi: 10.3390/insects13050474 (PMC9147879; doi:10.3390/insects13050474)
Supplement: Supplementary file 1 [file insects-13-00474-s001.zip › insects-1685171-supplementary.pdf]

Supplementary Materials

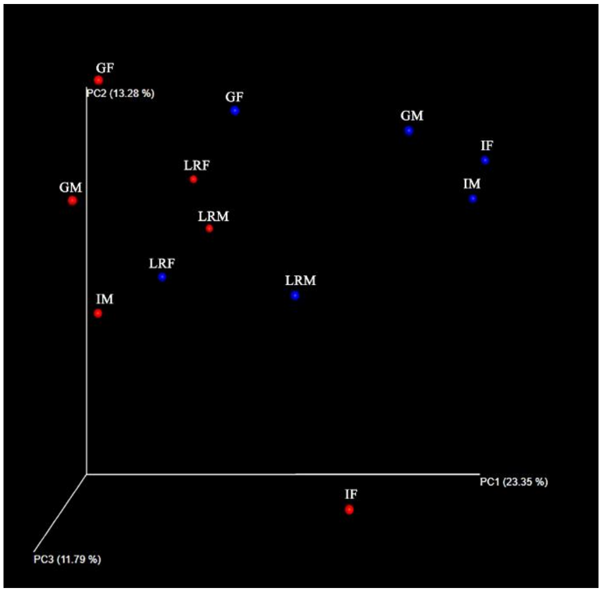

Figure S1. Principal Coordinates Analysis (PCoA) plots of samples colored according to different organs (gut = red; reproductive organs = blue). GM: Guatemala male; GF: Guatemala female; LRM: La Réunion male; LRF: La Réunion female; IM: ISPRA male; IF: ISPRA female.

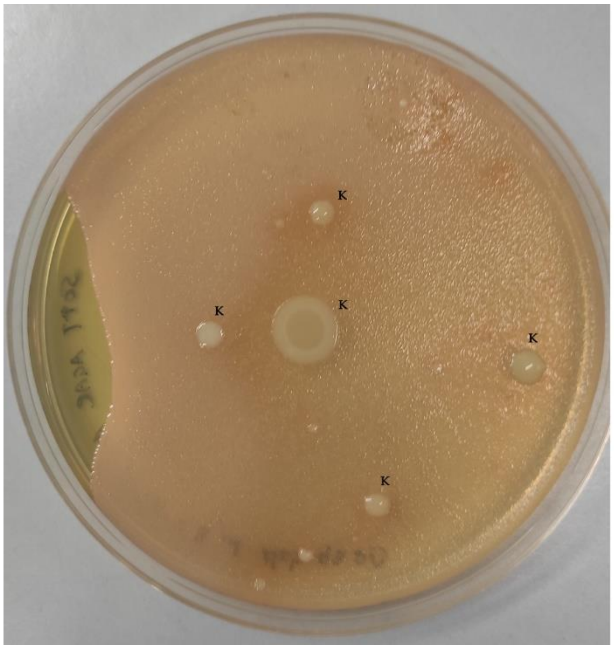

Figure S2. Antagonistic test of *Klebsiella oxytoca* against *Asaia* using the Agar diffusion assay. An aliquot from a *Asaia* culture was mixed on 0.7% agar and plated on GLY agar plate. Spots of 3 µl of *K. oxytoca* (K) were added on the surface. No zone of inhibition was recorded after 4 days of incubation at 26°C.

Table S1. % OTU Phyla in *C. capitata* strains.

	**Guat Gut M**	**Guat RO M**	**Guat Gut F**	**Guat RO F**	**La Re GUT M**	**La Re RO M**	**La Re GUT F**	**La Re OR F**	**ISPRA GUT M**	**ISPRA RO M**	**ISPRA GUT F**	**ISPRA RO F**
Taxonomy	%	%	%	%	%	%	%	%	%	%	%	%
Bacteria;Acidobacteria	0	0	0	0	0	0	0	0	0	0	0	0,2
Bacteria;Actinobacteria	0	9,2	0	1	0	7,9	0	1,1	0	19,3	0	28,2
Bacteria;Armatimonadetes	0	0	0	0	0	0	0	0	0	0,8	0	0
Bacteria;Bacteroidetes	0	0	0	0,7	0	2,7	0	0,5	0	6	0,1	2,3
Bacteria;Cyanobacteria	0	0	0	0	0	0	0	0	0	15	0	7,8
Bacteria;Deinococcus-Thermus	0	0	0	0,2	0	0	0	0	0	1,2	0	0,8
Bacteria;Firmicutes	0,2	1	0,4	1,7	0	2	0,1	0,1	0	2,6	0,1	1,9
Bacteria;Fusobacteria	0	0	0	0	0	0,2	0	0	0	0,2	0	0
Bacteria;Gemmatimonadetes	0	0	0	0	0	0	0	0	0	0,1	0	0
Bacteria;Proteobacteria	99,8	89,8	99,5	96,4	99,9	87,2	99,9	98,3	99,9	54,8	99,8	58,8
Other	0,2	0	0,5	0,9	0,1	0,2	0,1	0,6	0,1	1,1	0,2	1
Other	0,2	0	0,5	0,9	0,1	0,2	0,1	0,6	0,1	1,1	0,2	1

* Guat = Guatemala strain, La Re = La Réunion strain, ISPRA = ISPRA strain, RO = Reproductive organs, M = male, F = female.

Table S2. % OTU Genera in *C. capitata* strains.

	**Guat Gut M**	**Guat RO M**	**Guat Gut F**	**Guat RO F**	**La Re GUT M**	**La Re RO M**	**La Re GUT F**	**La Re RO F**	**La Re GUT M**	**La Re RO M**	**ISPRA GUT M**	**ISPRA RO M**	**ISPRA GUT F**	**ISPRA RO F**
**Taxonomy**														
Propionibacterium	0	9	0	1	0	6,9	0	1	0	6,9	0	15,9	0	20,7
Bacteroides	0	0	0	0,7	0	1,7	0	0,4	0	1,7	0	1,4	0	0
Hymenobacter	0	0	0	0	0	0	0	0	0	0	0	2,9	0	0,7
Chroococcidiopsis	0	0	0	0	0	0	0	0	0	0	0	14,9	0	7,6
Staphylococcus	0	0,3	0	0	0	0,2	0	0	0	0,2	0	0,4	0	1,5
Enterococcus	0,2	0,4	0,4	1,2	0	0	0,1	0	0	0	0	0	0	0
Streptococcus	0	0,2	0	0	0	1,8	0	0	0	1,8	0	0,5	0	0,4
Asaia	0,2	1,1	1,4	0,4	0,6	8	0,5	0,4	0,6	8	1	7,4	0,6	8
Gluconobacter	0,3	0,3	0,4	0,3	1	3,2	0,6	0,2	1	3,2	0,3	3,6	0,2	3,2
Sphingomonadales	0	0	0	0,3	0	0	0	0	0	0	0	1,7	0	1,3
Escherichia-Shigella	0	0,2	0	0	0	4,2	0	0	0	4,2	0	1,3	0	1
Klebsiella	90,8	81,6	85,7	69	97,7	65,4	87,5	1,8	97,7	65,4	96,6	34,4	98,2	33,9
Providencia	8	6	11,9	25,6	0,3	3,3	11	95,7	0,3	3,3	1,9	3,1	0,5	7,9
Other	1,2	2,3	1	3,2	1,3	5,5	1,5	1,5	1,3	5,5	0,5	13,4	1,8	14,9

* Guat = Guatemala strain, La Re = La Réunion strain, ISPRA = ISPRA strain, RO = Reproductive organs, M = male, F = female.
